# Epidemic of *Plasmodium falciparum* Malaria Involving Substandard Antimalarial Drugs, Pakistan, 2003

**DOI:** 10.3201/eid1511.090886

**Published:** 2009-11

**Authors:** Toby Leslie, Harpakash Kaur, Nasir Mohammed, Kate Kolaczinski, Rosalynn L. Ord, Mark Rowland

**Affiliations:** London School of Hygiene and Tropical Medicine, London, UK (T. Leslie, H. Kaur, K. Kolaczinski, R.L. Ord, M. Rowland); HealthNet TPO, Kabul, Afghanistan (T. Leslie, N. Mohammed)

**Keywords:** Malaria, epidemic, parasites, antimalarial drugs, Plasmodium falciparum, refugees, Pakistan, Afghanistan, vector-borne infections, research

## Abstract

Because of instability in eastern Afghanistan, new refugees crossed into the federally administered tribal areas of northwestern Pakistan in 2002. In 2003, we investigated an epidemic of *Plasmodium falciparum* malaria in 1 of the camps. Incidence was 100.4 cases/1,000 person-years; in other nearby camps it was only 2.1/1,000 person-years. Anopheline mosquitoes were found despite an earlier spray campaign. Documented clinical failures at the basic health unit prompted a drug resistance survey of locally manufactured sulfadoxine-pyrimethamine used for routine treatment. The in vivo failure rate was 28.5%. PCR analysis of the *P. falciparum* dihydrofolate reductase and dihyropteroate synthase genes showed no mutations associated with clinical failure. However, chemical analysis of the drug showed that it was substandard. As global incidence decreases and epidemics become more of a threat, enhanced quality assurance of control interventions is essential.

## Abstract 2

To prevent future epidemics, enhanced quality assurance is essential.

Epidemics make a major contribution to the global impact of malaria (*1*). Predisposing factors include level of disease endemicity, immune status of the population, changes in vector–person contact, unusual weather phenomena, conflict or mass population movement, and the capacity of public health systems to detect epidemics and respond (*1*). Epidemic malaria is more common in areas of mesoendemicity or hypoendemicity (*2*,*3*).

Detection and control require rapid reporting of reliable surveillance data and the capacity to analyze and interpret trends. An epidemic can be defined as a “sharp increase in the frequency of malaria transmission that exceeds by far the inter-seasonal variation normally experienced” (*4*). Distinguishing a seasonal increase from an epidemic may require comparison with historic data from the same locality (*1*,*5*), but such data may be unavailable or unreliable. This finding is particularly true for regions where conflict leads to population upheaval, creation of new settlements, and breakdown of health services (*6*,*7*). Adequate control requires rapid response using health information campaigns, reinforced diagnostic services, effective short-course drugs, preventative measures, and political commitment.

The Federally Administered Tribal Areas (FATA) of northwestern Pakistan are semiautonomous, tribally governed agencies running north–south along the western border with Afghanistan. These are areas of rugged terrain along a porous and unstable border (the Durrand Line). The region has been an area of complex emergency since 1979 and is currently unstable because of conflict between Pakistan’s armed forces and insurgents in FATA and the ongoing conflict across the Durrand Line in Afghanistan.

We describe an epidemic of *Plasmodium falciparum* malaria among Afghan refugees who in 2002 had fled to FATA after drought and war in the neighboring Afghan provinces of Nangahar, Paktia, and Khost. We report the causes and epidemiology of the outbreak and the health service response.

## Materials and Methods

### Study Area and Health Services

For 3 decades, FATA has hosted one of the largest refugee migrations, beginning with >3 million Afghans crossing the border to escape the conflict in Afghanistan (*8*). In response to the initial refugee crisis, >200 refugee camps were established under the mandate of the United Nations High Commissioner for Refugees (UNHCR) and the Pakistan Commissionerate for Afghan Refugees. Primary healthcare was provided by nongovernmental organizations through basic health units (BHUs) in each camp.

Ashgaroo camp was formed in 2002 in Khurram Agency, near the town of Parachinar, after drought and conflict in the neighboring Afghan provinces of Nangahar, Paktia, and Khost forced several thousand refugees to cross the border. Refugees were provided with tarpaulins and used locally acquired mud and straw to construct rudimentary shelters. The camp was situated in an area of semiarid grassland. A few kilometers to the east, the land is irrigated by the Khurram River, a tributary of the Indus River. Rice, wheat, and maize are grown in this region by the local Pakistani population. The UNHCR demographic surveillance system showed that the population of the camp varied between 8,000 and 10,000 (HealthNet TPO, 2005, unpub. data).

Malaria is seasonal in the region; ≈85% of cases are caused by *P*. *vivax* and the remainder by *P*. *falciparum* (*6*). An integrated malaria control program was run through the UNHCR network of clinics and field laboratories; technical support was provided by the nongovernmental organization HealthNet TPO. First-line treatments were sulfadoxine-pyrimethamine (SP) for patients with *P*. *falciparum* malaria and chloroquine for patients with *P*. *vivax* malaria.

### Analysis of Malaria Surveillance Data

Routine malaria surveillance data were the primary data source for analysis of the epidemic. These data were compiled each month by BHU staff in each refugee camp and transmitted through the health information system to provincial health authorities. Health services were free and most refugees used the BHUs. Outpatients with suspected malaria were referred to a microscopist for diagnosis. Patients with confirmed malaria were treated according to local guidelines with chloroquine for *P*. *vivax* and SP for *P*. *falciparum* or mixed infections. The quality assurance system, using blinded cross-checking of positive and negative slides, ensured a field microscopy accuracy >98% (*9*).

Case records entered in malaria registers consisted of age, sex, slide result, and treatment given. The total number of slides examined and the age and sex of persons with *P*. *vivax*, *P*. *falciparum*, and mixed infections were submitted each month to HealthNet TPO and collated by camp into monthly summaries of numbers of malaria cases, slide positivity rate, and incidence.

To compare with Ashgaroo data, 2 other datasets were accessed from the surveillance data of the Kurram Agency for 2002–2004. The first dataset was summary data for the camps of Bassoo and Old Bagzai which were established for new refugees at the same time as Ashgaroo. The second dataset was summary data for 6 camps established in Kurram in the early 1980s; these camps have longer established health services. All camps were similar in that they were occupied by Afghan refugees and situated in the same locality (Khurram Agency). The older camps had mud houses and a well-established health system. Newer camps were a mixture of tarpaulin shelters with mud walls, were more remotely situated, lacked electricity, and had a recently established health center.

Analysis of the epidemic compared 3 parameters: number of cases of malaria in persons who came to BHUs, slide positivity rate (no. cases positive for malaria/total no. slides examined), and incidence rate (no. cases/total person-months at risk). These parameters were estimated by using population data from UNHCR and passive surveillance data from camp BHUs.

### Monitoring of Drug Resistance

Monitoring of drug resistance was conducted in response to reports of treatment failure. Patients with microscopically confirmed *P*. *falciparum* infection gave informed consent and were enrolled in an in vivo drug resistance study. These patients were administered the same locally manufactured SP used by BHU health workers for routine treatment. The dose administered was based on weight of the patient and was noted on patient record forms. Patients were monitored for vomiting. Patients returned for consultation and collection of blood smears at weekly intervals for 42 days or at any time if symptoms recurred. Any absentee was followed up in their shelters and classified as lost to follow-up if absent for >2 consecutive days. Treatment failures in patients were defined by using criteria of the World Health Organization (WHO) (*10*); these patients were treated with mefloquine. A blood sample was obtained at the time of enrollment for detection of mutations in the *P*. *falciparum* dihydrofolate reductase (*Pfdhfr*) and dihyropteroate synthase (*Pfdhps*) genes, which are known to be associated with antifolate resistance (*11*,*12*).

An in vivo resistance study was conducted concurrently by HealthNet TPO in 3 long-established refugee camps 50–100 km north of the study site and situated outside the tribal areas. As in Ashgaroo, patients were enrolled if they had microscopically confirmed *P*. *falciparum* malaria but these patients were treated with SP (500 mg sulfadoxine and 25 mg pyrimethamine) (Fansidar; Roche, Basel, Switzerland) provided by WHO. Supervision was the same as in Ashgaroo, with a 42-day observation period. Slides from both trials were read by 2 microscopists and discordant slides were read by a third. In both studies, the primary outcome was defined as any malaria treatment failure, whether clinical or parasitologic, over the 42-day observation period. Simple proportions and univariate and multivariate logistic regression analyses were used to assess associations with failure, correcting for sex and age (STATA version 8; StataCorp., College Station, TX, USA).

### Drug Quality Evaluation

SP (Fansidar; Roche) and the locally manufactured SP were analyzed for quantity of active ingredient by using in vitro dissolution testing protocols according to procedures outlined in the United States Pharmacopeia and by high-performance liquid chromatography (HPLC). The test for content expresses the amount of active ingredient as a percentage of the label claim, and the test for dissolution determines the amount of active ingredient released and available for absorption (*13*).

Tablet dissolution was performed in the Pharma Test PT 017 dissolution apparatus (*Pharma Test* Apparatebau, Hainburg, Germany) and analyzed by using HPLC (*14*). Drug quality was assessed by comparing the amount of active ingredient in eluents of each dissolution sample against a known concentration of the standard for sulfadoxine and pyrimethamine after HPLC analysis.

### Vector Control

Entomologic investigation was conducted by space spray collection of mosquitoes conducted in 5 randomly selected compounds (animal sheds and sleeping areas) in Ashgaroo camp on 1 day. Windows were sealed and rooms were sprayed with a pyrethroid spray canister. Specimens were collected from white floor sheets and identified to species.

## Results

### Initial Outbreak Investigation and Response

In late June 2003, cases of *P*. *falciparum* malaria were reported in Ashgaroo camp. Because cases are not usually seen before mid-August, an investigation was mounted that strengthened case reporting and treatment guidelines. By August, the number of cases in Ashgaroo had increased above the usual level for the time of year. The epidemic response team directed to the camp confirmed a high vector density. A total of 717 anopheline specimens were collected from the 5 compounds, mostly from animal sheds. Of these specimens, 690 (96%) were *Anopheles subpictus*, 6 were *A*. *stephensi*, 14 were *A*. *culicifacies*, and data for 7 were not available. Anopheline larvae were found in several locations, mainly in borrow pits recently dug by the refugees for construction of mud shelters. Typically, borrow pits were several meters wide and deep and fed by water drained from the camps’ water tanks or from unseasonable rain, which maintained adequate water levels for mosquito breeding.

The epidemic response was to ensure adequate supplies of drugs, provide diagnostic and treatment services 24 hours per day, and apply larvicide to confirmed breeding sites. Insecticide-treated nets were made available at a highly subsidized price (UNHCR policy at the time). Active case detection was not carried out until October and November.

### Epidemic Trends

#### Malaria Cases

**Figure 1:**
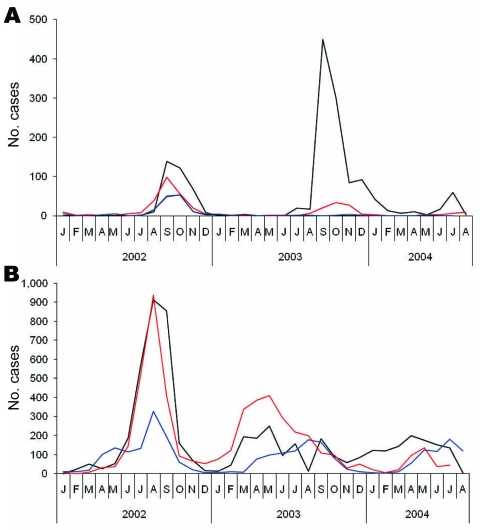
Number of cases of A) *Plasmodium falciparum* and B) *P*. *vivax* malaria in refugee camps in Khurram Agency, Pakistan, 2002–2004. Black lines indicate Ashgaroo camp, red lines indicate Bassoo and Old Bagzai camps combined, and blue lines indicate the remaining 6 older camps.

The trend in number of malaria cases recorded during 2002–2004 is shown in Figure 1. A steep increase in the number of *P*. *vivax* cases in the 3 new camps of Ashgaroo, Bassoo and Old Bagzai began in June 2002 at the beginning of the transmission season and peaked in August 2002 (Figure 1). In 2003, the increase in cases started much earlier, a normal occurrence because of delayed patency or relapse of cases from the previous year’s transmission. Insecticide spraying of Bassoo and Old Bagzai in 2003 appeared to reduce the number of *P*. *vivax* and *P*. *falciparum* cases that otherwise occurred that summer by curtailing transmission.

The *P*. *falciparum* malaria epidemic in Ashgaroo developed rapidly from 17 cases in August 2003 to 438 in September 2003. By November, the number of cases (82) had decreased to levels expected for that time of year. Using routinely reported mortality data as the indicator, we did not observe excess deaths in the camp. Few co-infections were recorded (1 in 2002 and 22 in 2003).

#### Malaria Incidence

**Figure 2:**
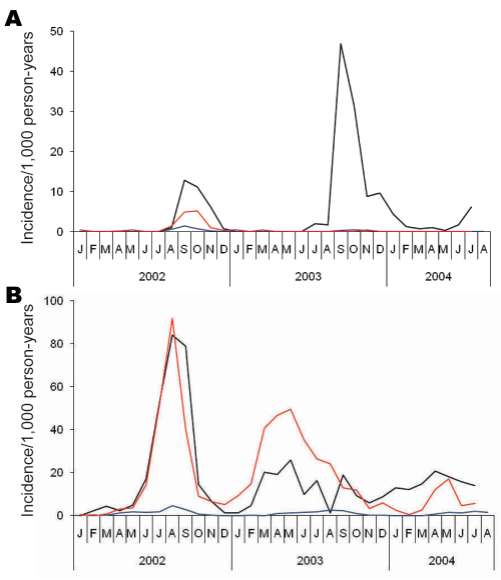
Incidence per 1,000 person-months of A) *Plasmodium falciparum* and B) *P*. *vivax* malaria in refugee camps in Khurram Agency, Pakistan, 2002–2004. Black lines indicate Ashgaroo camp, red lines indicate Bassoo and Old Bagzai camps combined, and blue lines indicate the remaining 6 older camps.

An unusually high incidence of *P*. *vivax* malaria was recorded in the 3 newly established refugee camps (Ashgaroo, Old Bagzai, and Bassoo) in the summer of 2002 (Table 1, Figure 2). Incidence of *P*. *vivax* (269 cases/1,000 person-years) and *P*. *falciparum* malaria (32 cases/1,000 person-years) was 15.6× higher (95% confidence interval [CI] 14.7–16.7×] for *P*. *vivax* and 6.9× higher (95% CI 5.9–8.1×) for *P*. *falciparum* than in the 6 remaining camps. The range in annual incidence of *P*. *vivax* malaria in the 3 new camps was similar (130–296 cases/1,000 person-years). In contrast, the annual incidence of *P*. *falciparum* malaria was similar in Ashgaroo (32 cases/1,000 person-years) and Bassoo (22 cases/1,000 person-years) but was ≈10× lower than the annual incidence of *P*. *vivax* malaria (269 cases/1,000 person-years).

**Table 1:** Annual incidence of *Plasmodium vivax* and *P*. *falciparum* malaria per 1,000 population in camps in Khuram Agency, Pakistan, 2002–2003

Camp	2002			2003	
	*P. vivax*	*P. falciparum*		*P. vivax*	*P. falciparum*
Ashgaroo	268.9	32.3		152.3	100.4
Bassoo	296.1	22.0		283.6	1.1
Old Bagzai	130.6	3.5		275.3	4.2
Bassoo and Old Bagzai	226.5	14.2		280.9	2.1
Remaining 6 camps	15.9	3.4		12.5	1.4

Tents and houses were sprayed with insecticide before the onset of the 2003 malaria transmission season. During 2003, a total of 963 *P*. *falciparum* malaria cases were recorded in Ashgaroo (incidence 100.4/1,000 person-years). The incidence rate ratio relative to the previous year was 3.1 (95% CI 2.8–3.5). Compared with rate ratios for camps at Bassoo and Old Bagzai, the incidence rate ratio in Ashgaroo was 48.8 (95% CI 30.3–84.1). Compared with rate ratios for the 6 long-established camps, the ratio was 72.1 (95% CI 58.5–89.7). The incidence of *P*. *falciparum* malaria in Ashgaroo was only marginally lower than that for *P*. *vivax* malaria (*P*. *falciparum* 100.4/1,000 person-years vs *P*. *vivax* 142.6/1,000 person-years), whereas the *P*. *vivax* malaria:*P*. *falciparum* malaria incidence rate ratio for the region is usually of the order of 5:1.

#### Slide Positivity Rate

**Figure 3:**
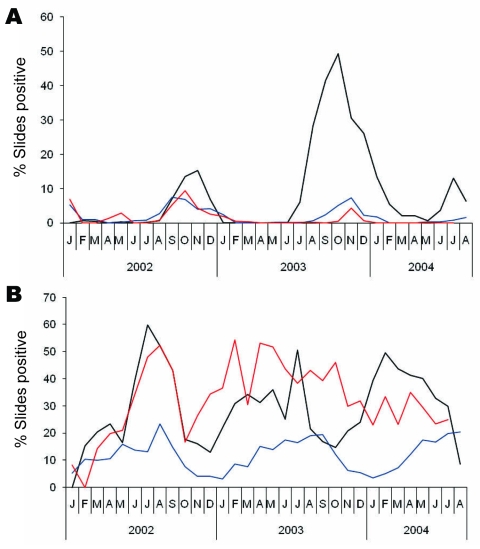
Slide positivity rate (% slides positive) for A) *Plasmodium falciparum* and B) *P*. *vivax* malaria in refugee camps in Khurram Agency, Pakistan, 2002–2004. Black lines indicate Ashgaroo camp, red lines indicate Bassoo and Old Bagzai camps combined, and blue lines indicate the remaining 6 older camps.

The slide positivity rate provides an opportunity to measure the proportion of febrile illness attributable to malaria. The advantage of this rate over estimates of incidence rate is that trends are unaffected by inaccuracies or changes in population. During epidemics, malaria positivity rates show a sudden increase. *P*. *viv*ax positivity rates for the new camps in 2002 and 2003 were only 2.9× higher than for long-established camps (Figure 3), and *P*. *falciparum* positivity rates for the new camps of Ashgaroo (4.7%) and Bassoo (3.6%) in 2002 were also similar to those for the older camps (2.9%). However, in 2003, the *P*. *falc*iparum positivity rate showed a marked increase for Ashgaroo (18.8%) and reached 50% positivity during the peak of the epidemic in October, a rate 9× higher than for the neighboring camps.

### Evaluation of Drug Efficacy

In September 2003, staff reported that many patients were returning with recurrent malaria after treatment with SP. These cases were confirmed microscopically and treated with mefloquine, the second-line treatment. Using the in vivo drug resistance protocol, we recruited 169 patients with malaria cases at the clinic and who were treated with SP (Table 2). Selection was based on clinical symptoms, malaria parasitemia, and granting of informed consent. Eighteen (10.6%) patients were lost to follow-up. Of those completing the study, treatment was not successful in 43 (28.5%) of 151 patients (Table 3). The comparison group showed a failure rate for treatment with SP from WHO of only 10% (4/40) (χ^2^ = 5.8, p = 0.02) (Table 3).

**Table 2:** Enrollment characteristics of population from in vivo drug resistance survey conducted in Ashgaroo and concurrent drug study in Yakaghund camp, Pakistan, October 2003

Characteristic	Ashgaroo	Yakaghund
No. (%) patients	169 (79.0)	45 (21.0)
% Male sex	41.8	66.7
Mean age, y (SD)	13.3 (11.9)	19.4 (13.2)
No. (%) lost to folllow-up	18 (10.7)	5 (11.1)

**Table 3:** Frequency of treatment failure for infection with *Plasmodium falciparum* or *P. vivax*, by group, sex, and age, Pakistan, October 2003*

Characteristic	Failure rate (%)	Crude OR (95% CI)	Adjusted OR (95% CI)
Group			
Comparison group	4/40 (10.0)	1	1
Ashgaroo	43/151 (28.5)	3.6 (1.2–10.7)	3.2 (1.0–10.1)
Sex			
M	27/90 (30.0)	1	1
F	20/99 (20.2)†	0.6 (0.3–1.2)	0.5 (0.3–1.2)
Age group, y			
0–5	16/40 (40.0)	1	1
6–10	21/72 (29.2)	0.6 (0.3–1.4)	0.6 (0.3–1.1)
11–20	4/33 (12.2)	0.2 (0.1–0.7)	0.3 (0.1–1.0)
>20	6/46 (13.0)	0.2 (0.1–0.6)	0.3 (0.1–0.8)

*OR, odds ratio; CI, confidence interval. †Results exclude losses to follow-up.

PCR was conducted on specimens from 81 persons; 37 samples were taken on the day of enrollment and day of treatment failure and 35 had no matching enrollment sample. All patients in whom treatment was not successful were analyzed for merozoite surface protein 2; all had recrudescent infections and not reinfections (5 samples did not yield a product). Analysis also assessed 5 loci on the *Pfdhfr* gene at positions 16, 50/51, 59, 108, and 164, and 4 loci on the *Pfdhps* gene at positions 436/437, 540, 581, and 613. Among samples matched with treatment failures, only 1 specimen showed any mutation (A16V) in the *Pfdhfr* gene. Single mutations were found in 6 of the baseline samples, but none of these polymorphisms on their own have been associated with clinical failure (*11*,*12*).

Samples of SP tablets used for routine treatment and for the drug resistance study were found to be substandard. WHO has defined substandard drugs as “the genuine drug products which do not meet quality specifications set for them. If a drug, upon laboratory testing in accordance with the specifications it claims to comply with fails to meet the specifications, then it is classified as a substandard drug.” By 30 minutes of dissolution, only 52% sulfadoxine (0.156 mg/mL, n = 9) and ≈16% pyrimethamine (0.0025 mg/mL, n = 9) was detected against the stipulation that 60% (sulfadoxine 0.3 mg/mL and pyrimethamine 0.015 mg/mL) should be dissolved at this point. The dissolution profile for Fansidar met stated tolerance limits, but the locally manufactured generic tablets did not. Content analysis by HPLC showed that the generic tablets contained greater amounts of correct active ingredients (126% sulfadoxine/tablet and 128% pyrimethamine/tablet) than stated on the packaging. Because they failed to meet the criteria for dissolution tolerance, the drugs would not be released at the required dose (*13*).

## Discussion

The malaria epidemic in Pakistan was intense, short-lived, and controlled by a series of active and passive interventions. Strengthening of health services, active case detection and treatment, larviciding of anopheline breeding sites, and distribution of insecticide-treated nets each played their part, although separating their individual contributions is not possible. The onset of cooler weather in November brought an end to transmission. That no excess deaths were recorded is surprising, especially with substandard first-line treatment. The well-trained health staff, a well-run and close-at-hand clinic offering free treatment, an effective second-line drug, and public awareness likely contributed to this absence of excess deaths.

A combination of factors propagated the epidemic. Digging of borrow pits provided a new habitat for vector breeding. Epidemiologic evidence suggests failure of the spray campaign. Although the Bassoo and Old Bagzai spray campaigns appear to have curtailed transmission, the failure in Ashgaroo may have been caused by several factors. Insecticide resistance would enable vector survival, but this seems unlikely because malaria was well controlled in the other sprayed camps and resistance to pyrethroids has not been observed in this region. Effective implementation of spray campaigns requires quality-assured insecticide, thorough training and supervision of spray teams, and checks on operations. The third factor may have been deficient in Ashgaroo.

In the year before the epidemic, chloroquine was used as first-line treatment despite high resistance levels (*15*). This use would enable persistence of gametocytemia in some persons and a reservoir of infection (*16*). Evidence shows that gametocytes can persist for 1 year after infection (*17*). Submicroscopic gametocytemia detected by PCR indicates that the gametocyte reservoir is considerably higher than previously thought in areas of low or seasonal transmission (*18*,*19*).

In the Pakistan–Afghanistan region, only 1 epidemic has been reported recently, an outbreak of high-altitude malaria in Bamian, Afghanistan (*20*). There are similarities between that epidemic and the current one; cases were reported in June–August, when unusual environmental conditions enabled vector breeding, and population movement of infected refugees provided a source of cases into an area previously unaffected by malaria. This outbreak was circumscribed by the immune status of the affected population (nonimmune migrants) and the relative isolation of the camp.

*An*. *subpictus* mosquitoes were previously identified in the region and are competent vectors in Sri Lanka (*21*,*22*). Two studies in the Pakistan Punjab recorded peak abundance of *An*. *subpictus* mosquitoes in late summer and fall; the species occurred sympatrically with *An*. *stephensi* mosquitoes (*23*,*24*). Historically, *An*. *subpictus* mosquitoes are not a major vector in the Pakistan Punjab (*25*). In eastern Afghanistan, Rowland et al. (*9*) identified *An*. *stephensi* mosquitoes and to a lesser extent *An*. *culicifacies* mosquitoes as the main vectors; few *An*. *subpictus* mosquitoes were found by an 18-month surveillance study, and none were positive for circumsporozoite protein. *An*. *subpictus* mosquitoes are known to breed in muddy pools and borrow pits and later in the season than other potential vectors (*24*,*25*). Although the present study provides some evidence for *An*. *subpictus* being a more common vector than previously thought, it is likely that *An*. *stephensi* and *An*. *culicifacies*, the primary vector species (*9*), which were also recorded in low numbers during the surveys, played a part in transmission earlier in the summer during June and July. By September, the densities of these 2 vectors may have waned, as recorded in other longitudinal studies in eastern Afghanistan and Pakistan Punjab, but during the main transmission period were likely to have been more abundant (*9**,**23*).

Few mixed infections were seen. A recent metaanalysis (*26*) showed a negative association between *P*. *falciparum* and *P*. *vivax* but noted considerable heterogeneity related to prevalence of infection. Our data, which showed fewer *P*. *vivax* cases than expected in the epidemic period, indicated a suppressive effect of *P*. *falciparum* on *P*. *vivax*, as recently observed during a vector control campaign that controlled *P*. *falciparum* transmission but led to an increase in relapsed *P*. *vivax* cases (*6*).

The assessment of in vivo drug resistance was conducted in anticipation that a problem with SP resistance rather than substandard local drugs would be uncovered. Further assessments involving molecular characterization of SP resistance genes, drug quality testing, and in vivo surveys using standardized SP established that substandard drugs were the cause of what initially appeared as resistance. The drugs used routinely in the camp were procured centrally and distributed through local government organizations. A substandard drug in wide circulation will inevitably contribute to disease transmission and was undoubtedly a factor contributing to this epidemic. The substandard drugs were procured as a response to shortages. In surveys of other camps, standard SP showed a 90% cure, which indicated that low-level resistance may be an emerging problem in the study region. A distinction should be made between the more easily remedied use of poorly manufactured substandard drugs reported and the alarming growth of counterfeit drugs in Southeast Asia (*27*).

As the global prevalence of malaria decreases because of initiatives to control or eliminate the disease, more areas will become mesoendemic or hypoendemic for malaria and detection and control of epidemics will acquire greater attention. Elimination will not be easy in areas of complex emergency and population movement, and the uncontrolled production or use of substandard drugs will only add to the problem. Mechanisms to ensure quality of interventions are essential.
